# Draft Genome Sequence of Mycoplasma arginini Strain NGR_2017

**DOI:** 10.1128/genomeA.00577-18

**Published:** 2018-06-28

**Authors:** J. A. Adeniji, T. O. C. Faleye, O. M. Adewumi, O. A. Olayinka, E. Donbraye, B. Oluremi, U. E. George, O. A. Arowolo, E. C. Omoruyi, M. I. Ifeorah, A. Akande

**Affiliations:** a Department of Virology, College of Medicine, Faculty of Basic Medical Sciences, University of Ibadan, Ibadan, Nigeria; b Department of Microbiology, Faculty of Science, Ekiti State University, Ado-Ekiti, Nigeria; c Department of Medical Microbiology and Parasitology, Obafemi Awolowo University, Ile-Ife, Nigeria; d Department of Pharmaceutical Microbiology, Faculty of Pharmacy, University of Ibadan, Ibadan, Nigeria; e Viral Vaccines Production Division, National Veterinary Research Institute, Vom, Plateau State, Nigeria; f Institute of Child Health, College of Medicine, University of Ibadan, Ibadan, Nigeria; g Department of Medical Laboratory Sciences, Faculty of Health Sciences and Technology, University of Nigeria, Nsukka, Nigeria; h WHO National Polio Laboratory, University of Ibadan, Ibadan, Nigeria

## Abstract

We present the draft genome of Mycoplasma arginini strain NGR_2017. This strain was recovered in Nigeria from cell culture in 2017. The assembly contains 620,555 bp in 12 contigs. It contains 561 coding sequences, 34 RNAs (29 tRNAs, 4 rRNAs, and 1 transfer-messenger RNA [tmRNA]), and a >26-kb integrative and conjugative element.

## Abstract (precis)

We present the draft genome of Mycoplasma arginini strain NGR_2017. This strain was recovered in Nigeria from cell culture in 2017.

## GENOME ANNOUNCEMENT

Mycoplasma arginini is a wall-less bacterium with zoonotic potential that belongs to the class Mollicutes. It has been recovered, with various presentations, from sources ranging from cell cultures (1) through animal specimens (2) to immunocompromised individuals (3). Here, we present the first draft genome sequence of M. arginini from Nigeria, isolated from cell culture supplemented with streptomycin and penicillin (4, 5).

Total DNA was extracted from cell culture supernatant of both RD and L20B cell lines using a viral RNA/DNA extraction kit (Jena Bioscience, Jena, Germany). DNA was shipped to a commercial facility (MR, Texas, USA) where whole-genome sequencing and contig assembly were done. Briefly, a sequencing library was prepared using a Nextera DNA sample preparation kit (Illumina) following the manufacturer's instructions, and sequencing was done using the HiSeq system (Illumina). After quality trimming and error correction were completed, 359,032 quality reads in 12 contigs contained the draft M. arginini genome. The genome contains 620,555 bp with a GC content of 26.1%. Genome completeness was estimated as 96.6% using CheckM (6). Annotation was performed using RAST (7–9), and M. arginini strain NGR_2017 was predicted to contain 561 coding sequences and 34 RNAs, including 29 tRNAs, 1 transfer-messenger RNA (tmRNA), and 4 rRNAs (2 small- and 2 large-subunit rRNAs).

Compared to the reference M. arginini genome in GenBank (Japan 2008), strain NGR_2017 has lost most of the type I restriction modification enzymes, prophage sequences, and CRISPR-associated proteins. The genome of the NGR_2017 strain has also been significantly reorganized. Furthermore, M. arginini NGR_2017 has acquired one (>26 kb) integrative and conjugative element (ICE) (10) with 23 coding sequences (which was not present in the Japan 2008 strain). To the best of our knowledge, this represents the first description of ICE in M. arginini. This ICE encodes, among other genes, a type IV secretory protein conjugative transfer family protein, serine hydratase, DNA primase, site-specific DNA modification methyltransferase, integrative conjugal element protein ICEF-IIB (protein 22), E3 ubiquitin protein ligase, and putative lipoproteins, metallo-endopeptidase, and other conserved ICE parts.

Within the genomic backbone, just like the Japan strain, NGR_2017 has a multidrug resistance ABC transporter ATP-binding and permease protein. Whether this contributes in any way to the ability of NGR_2017 to grow in the presence of streptomycin is currently not clear.

### Accession number(s).

This whole-genome shotgun project has been deposited at DDBJ/ENA/GenBank under the accession number QFDN00000000. The version described in this paper is version QFDN01000000.
